# Exploring spatiotemporal pattern in the association between short-term exposure to fine particulate matter and COVID-19 incidence in the continental United States: a Leroux-conditional-autoregression-based strategy

**DOI:** 10.3389/fpubh.2023.1308775

**Published:** 2023-12-22

**Authors:** Shiyi Liu, Shuming Ji, Jianjun Xu, Yujing Zhang, Han Zhang, Jiahe Liu, Donghao Lu

**Affiliations:** ^1^Department of Hospital Infection Management, Chengdu First People’s Hospital, Chengdu, China; ^2^Department of Clinical Research Management, West China Hospital, Sichuan University, Chengdu, China; ^3^School of Mathematics and Statistics, University of Melbourne, Melbourne, VIC, Australia; ^4^Faculty of Art and Social Science, University of Sydney, Sydney, NSW, Australia

**Keywords:** spatiotemporal heterogeneity, PM_2.5_ exposure, vaccination modification, spatial dependence, conditional autoregression

## Abstract

**Background:**

Numerous studies have demonstrated that fine particulate matter (PM_2.5_) is adversely associated with COVID-19 incidence. However, few studies have explored the spatiotemporal heterogeneity in this association, which is critical for developing cost-effective pollution-related policies for a specific location and epidemic stage, as well as, understanding the temporal change of association between PM_2.5_ and an emerging infectious disease like COVID-19.

**Methods:**

The outcome was state-level daily COVID-19 cases in 49 native United States between April 1, 2020 and December 31, 2021. The exposure variable was the moving average of PM_2.5_ with a lag range of 0–14 days. A latest proposed strategy was used to investigate the spatial distribution of PM_2.5_-COVID-19 association in state level. First, generalized additive models were independently constructed for each state to obtain the rough association estimations, which then were smoothed using a Leroux-prior-based conditional autoregression. Finally, a modified time-varying approach was used to analyze the temporal change of association and explore the potential causes spatiotemporal heterogeneity.

**Results:**

In all states, a positive association between PM_2.5_ and COVID-19 incidence was observed. Nearly one-third of these states, mainly located in the northeastern and middle-northern United States, exhibited statistically significant. On average, a 1 μg/m^3^ increase in PM_2.5_ concentration led to an increase in COVID-19 incidence by 0.92% (95%CI: 0.63–1.23%). A U-shaped temporal change of association was examined, with the strongest association occurring in the end of 2021 and the weakest association occurring in September 1, 2020 and July 1, 2021. Vaccination rate was identified as a significant cause for the association heterogeneity, with a stronger association occurring at a higher vaccination rate.

**Conclusion:**

Short-term exposure to PM_2.5_ and COVID-19 incidence presented positive association in the United States, which exhibited a significant spatiotemporal heterogeneity with strong association in the eastern and middle regions and with a U-shaped temporal change.

## 1 Introduction

Fine particulate matter (PM_2.5_) indicates the particulate matter whose diameter is smaller than 2.5 μm. PM_2.5_ is a common air pollutant that has been identified as a significant environmental factor affecting the respiratory, immune, and circulatory systems (1). The main mechanisms include increasing oxidative stress and inflammatory responses and causing injury to lung circulation and endothelial dysfunction (2, 3). In viral lung and respiratory infections, PM_2.5_ is believed to play a role in various virus life cycle stages, including inhibiting mucociliary clearance, altering viral receptors, inhibiting antiviral interferon production, and enhancing epithelial permeability (4, 5).

The COVID-19 pandemic is caused by severe acute respiratory syndrome coronavirus 2 (SARS-CoV-2) and has been prevalent worldwide for approximately 3 years (6). It is the largest global public health crisis faced by mankind since the Second World War (7). Due to its high infectiousness and strong ability to evade the immune system, COVID-19 has already and is bringing heavy economic and health burdens to humanity (8).

Since the outbreak of this pandemic, the concentration of air pollutants has changed significantly from before the outbreak, and the impact of pollutant exposure on the COVID-19 epidemic is widely concerned (9, 10). Many studies have reported that short-term exposure to PM_2.5_ may increase the risk of COVID-19 (11, 12). However, considerable heterogeneity between regions was observed in these studies. For instance, a study in London, the United Kingdom, revealed that a 1 μg/m^3^ increase in PM_2.5_ is associated with a 1.1% increase in COVID-19 cases (13); a study in California, the United States, revealed that a 1 μg/m^3^ increase in PM_2.5_ is associated with a 0.2% increase in COVID-19 cases (14). Moreover, another study conducted in the northwestern and southern regions of the United States revealed a positive association between PM_2.5_ exposure and COVID-19 incidence, however, its subgroup analysis exhibited significant differences between the regions (15). Sitian Zang et al. also identified a strong heterogeneity in the associations between PM_2.5_ and COVID-19 incidence among 35 observational studies (heterogeneity *I*^2^ = 94.2%; *p* < 0.05) (16).

The heterogeneity may have been caused by different analysis methods (17); however, it is more likely to result from different effect-modifying factors, including political and social factors, meteorological conditions, and population density (18). These factors are significantly different between regions and study periods. Owing to the existence of substantial heterogeneity, evaluating the overall association in a large-scale area would ignore local variations, potentially leading to inaccurate association and even inappropriate public health decision making in some local regions (19).

The United States is one of the countries suffering most from COVID-19 pandemic. As of December 2021 end, a total of more than 52 million COVID-19 cases had been recorded in the United States, of which 824 thousand have died. Because of the environmental and political differences, the effect-modifying factors and the epidemic prevention and control polices for COVID-19 considerably vary across different epidemic periods and states (20). These factors may have resulted in a substantial spatiotemporal heterogeneity in the association between COVID-19 incidence and PM_2.5_. Therefore, from a public health point, it is greatly important to investigate this heterogeneity in the United States. Owing to the abundant medical and health resources as well as the accurate case information registration and management system, high-quality and easily available monitoring COVID-19 data make it available to explore the spatiotemporal heterogeneity of PM_2.5_-COVID-19 association in the United States.

The classic method for investigating the heterogeneity of associations were the meta-analysis-based two-stage strategy by Gasparini et al.’s work (21–23). In the first stage, time-series generalized additive models (GAMs), which share a model structure, were independently constructed to estimate a preliminary association for each region; then, meta-analysis was used to pool the associations by borrowing information and explore the potential effect modifiers by including region-level factors. However, this strategy does not consider the commonly existed spatial autocorrelation, thus obtaining inaccurate spatial distributions of association and substantially inflating the false positive error in identifying the effect modifiers (24–26). To address this limitation, the Leroux conditional autoregressive (LCAR)-based strategy was proposed in the recent (27), which cannot only sufficiently utilize the spatial autocorrelation to improve the result, but also can explore the spatiotemporal heterogeneity of association by combing with the time-varying GAM.

In this work, based on the daily numbers of recorded COVID-19 cases and the daily concentration of PM_2.5_, we used the advanced LCAR-based analytical strategy to elucidate the spatiotemporal heterogeneity in the short-term association between PM_2.5_ exposure and COVID-19 incidence, explore its temporal change, and identify the potential causes for this heterogeneity. Our research may assist the decision-makers to establish cost-effective pollution-related policies for a specific location and epidemic stage, as well as, help to understand the temporal change in the association between air pollutants like PM_2.5_ and a sudden and severe infectious disease like COVID-19.

## 2 Data

The study region includes 49 states in the continental United States, where the special district (Columbia) was also deemed as a state for writing convenience. All the state names were abbreviated as two capital letters from the full names for clarity. Supplementary Table S1 and Supplementary Figure S1 gives a detailed introduction about the abbreviation and full names and the geographic locations of states.

### 2.1 Data collection and processing

Between April 1, 2020 and December 31, 2021, the daily numbers of COVID-19 cases and the vaccination data at the state level were gathered from the Centers for Disease Control and Prevention (CDC) of United States (28). The vaccination data included two kinds of rates. One is the population percentages of receiving at least one dose (PP1V), the other is the percentages of population with full vaccination (PPFV). The daily PM_2.5_ concentrations with a 0.75^°^ × 0.75^°^ resolution were obtained from the fourth-generation global reanalysis of ECMWF regarding atmospheric composition (29). Temperature, air pressure, wind speed, and other meteorological variables were gathered from 64,346 environmental monitoring stations using (30).^1^ The 24th United States census, which was conducted in 2021, provided the county-level population statistics.^2^ The Bureau of Labor Statistics^3^ provided the economic data.

We applied a kriging interpolation method, incorporating with population weighting, to acquire a more accurate daily exposure to PM_2.5_ by considering the population’s uneven spatial distribution. First, PM_2.5_ concentrations and high-resolution meteorological data were obtained using the ordinary kriging approach with a 1 km × 1 km grid resolution (31). Then, the county-level concentration of PM_2.5_ was obtained by average all the grid value for a specific county. Likewise, the daily values of meteorological factors were also computed for each state, including wind speed, air pressure, and temperature.

## 3 Methods

### 3.1 GAM-LCAR strategy: characterizing the spatial distribution In association

In the first stage, as in the classic strategy, for each state, a common analytic process was used to obtain a rough estimation for the PM_2.5_-COVID-19 association. Specifically, time-series GAMs, which share a model structure, were built independently for each state, which can be written as:


$$Y_{t}\sim \mathrm{Quasi}-\mathrm{Poisson}\left(u_{t}\right)\text{,}$$

(1) $$\begin{array}{l} \ln\left(u_{t}\right)=\alpha +\mathrm{\beta Pollu}\tan t_{t}+s_{1}\left(Temp_{t}\right)+s_{2}\left(AP_{t}\right)+\mathrm{\zeta Win}d_{t}\\ +s_{3}\left(Time_{t}\right)+\mathrm{Auto}.term_{t}+\mathrm{DO}W_{t}+\mathrm{Holida}y_{t}\text{,} \end{array}$$

where Quasi-Poisson distribution was used to characterize the potential overdispersion. $Y_{t}$ refers to the daily number of COVID-19 cases at time t for a specific state and $E\left(Y_{t}\right)\text{≡}u_{t}$. The term *α* is the intercept and β measures the intensity of association. $\mathrm{Pollutan}t_{t}$ refers to the exponential moving average (EMA) of PM_2.5_ where a lag range of 0–14 days was selected (32, 33) by minimizing the generalized cross-validation score (Supplementary Figure S2). Because meteorological factors, such as pressure, temperature and wind speed, may confound the PM_2.5_-COVID-19 association (34), in Equation (1) we also adjust for such confounding effects by using $s_{1}\left(Temp_{t}\right)$, $s_{2}\left(AP_{t}\right)$ and $\mathrm{\zeta Win}d_{t}$. Among them, $s_{1}\left(Temp_{t}\right)$ and $s_{2}\left(AP_{t}\right)$ were characterized by natural cubic spline functions where the degrees of freedom (df) were set as 4 and 3, respectively; $\mathrm{Win}d_{t}$ is the EMA of wind speed where the lag range is set as 0–14 days. The term $s_{3}\left(Time_{t}\right)$, a natural cubic spline function whose df is 10 per year, was used to characterize the long-term trend. All the mentioned df values and the employed confounders forms were determined by minimizing the Bayesian information criterion (BIC). Because COVID-19 presented strong infectivity, which may result in a temporal dependence between $Y_{t}$ and $Y_{t-1}$, we used an autoregressive term, $\mathrm{Auto}.term_{t}$, to reclaim the temporal independence among errors. In addition, $\mathrm{DO}W_{t}$ and $\mathrm{Holida}y_{t}$ were used to adjust for the effects from weekends and holidays, respectively. Supplementary Table S2 and Supplementary Figure S3 provided the detailed information on parameter selections.

In the second stage, let ${\hat{\beta }}_{i}$ be the estimated association from the first stage in state i, and ${\hat{\sigma }}_{i}$ be the corresponding standard error. Then, based on ${\hat{\beta }}_{i}$ and ${\hat{\sigma }}_{i}$, we constructed a LCAR model to smoothen the parameters for a more accurate spatial distribution of association (35). The LCAR model was established as:

$${\hat{\beta }}_{i}\sim N\left(\beta_{i},{\hat{\sigma }}_{i}^{2}\right)\text{,}$$

$$\beta_{i}=\eta +\xi_{i}\text{,}$$

(2) $$\xi \sim MN\left(0,{\left[\tau W\right]}^{-1}\right)\text{,}$$

(3) $$W=\rho R+\left(1-\rho \right)I\text{,}$$

where $\beta_{i}$ refers to the true association in state i. The term η reflects the average association across the 49 states. $\xi_{i}$ is the ith element of ξ which reflects the between-state heterogeneity in Equation (2). N(·) is the univariate normal distribution and MN(·) is the multivariate normal distribution. The intensity of spatial heterogeneity is measured by the precision parameter τ, a larger value of which indicates a smaller heterogeneity. W is the Leroux prior which characterizes the spatial dependence (35) whose intensity is measured by ρ. The symmetric matrix R is constructed according to the spatial adjacent relationship among states (35). We used the Integrated Nested Laplace Approximations (36, 37) to estimate the parameters. According to the pooled estimation for $\beta_{i}$, we obtained the spatial distribution of PM_2.5_-COVID-19 association.

### 3.2 Time-varying GAM-LCAR strategy: characterizing the temporal change of association and examining the effect modifiers

Because various SARS-CoV-2 variants have different infectivity and the potential effect-modifying factors, e.g., vaccination rate and variations in epidemic prevention policies as well as economic factors, vary over time; the associations between PM_2.5_ exposure and COVID-19 incidence may exhibit temporal heterogeneity, which has been found in the temperature-COVID-19 association (27). Therefore, by combining the time-varying GAM with the LCAR model, a modified time-varying strategy was further built to explore the temporal change of association and identify the potential effect modifiers.

Specifically, in the first stage, time-varying GAMs, which share a model structure, were independently built by incorporating a nonlinear interaction between PM_2.5_ exposure and time variable into Equation (1). For each state, we construct the time-varying GAM as follows:


(4) $$\begin{array}{c} \ln\left(u_{t}\right)=\alpha +s_{1}\left(Temp_{t}\right)+s_{2}\left(AP_{t}\right)+\mathrm{\zeta Win}d_{t}+s_{3}\left(Time_{t}\right)\\ \;+\mathrm{Auto}.term_{t}+\mathrm{DO}W_{t}+\mathrm{Holida}y_{t}\\ \;+s_{4}\left(Time_{t}\right)*\mathrm{Pollu}\tan t_{t}\text{,} \end{array}$$

where $s_{4}\left(Time_{t}\right)$ indicates the linear PM_2.5_-COVID-19 association at time *t*, i.e., $s_{4}\left(Time_{t}\right)=\beta_{t}$ in Equation (4). To ensure the temporal change of association to be continuous and flexible, we used a natural cubic spline function of time to characterize the temporal change, i.e., $s_{4}\left(Time_{t}\right)$ is set as a natural cubic spline function with df of 3. Then, at each time point t, like in Section 3.1, a LCAR model was built independently to pool the association parameters from time-varying GAMs for obtaining the time-specific PM_2.5_-COVID-19 associations.

To explore whether vaccination, temperature and economic factors significantly modifying the PM_2.5_-COVID-19 association, we furtherly constructed a spatiotemporal LCAR model as follow:


$${\hat{\beta }}_{\mathrm{it}}\sim N\left(\beta_{\mathrm{it}},{\hat{\sigma }}_{\mathrm{it}}^{2}\right)\text{,}$$

(5) $$\beta_{\mathrm{it}}=\eta +\theta x_{\mathrm{it}}+\xi_{i}+\gamma_{t}\text{,}$$

$$\xi \sim MN\left(0,{\left[\tau W\right]}^{-1}\right)\text{,}$$

$$\gamma_{t}\;|\gamma_{t-1}\sim N\left(\gamma_{t-1},\sigma_{\gamma }^{2}\right)\text{,}$$

Where ${\hat{\beta }}_{\mathrm{it}}$ is the estimated parameters in Equation (4) for state i. Leroux prior was used to characterize the spatial autocorrelation between $\xi_{i}$s as in Equation (3). Random walk prior was selected to characterize the temporal autocorrelation between $\gamma_{t}$s. The term $x_{\mathrm{it}}$ is a vaccination, temperature, or economic-related predictor, and θ measures the linear intensity of the effect modification. To explore the nonlinear effect modification, we also used the natural cubic spline function with a *df* of 3 to substitute the term $\theta x_{\mathrm{it}}$ in Equation (5).

## 4 Results

### 4.1 Descriptive analysis

**Figure 1:**
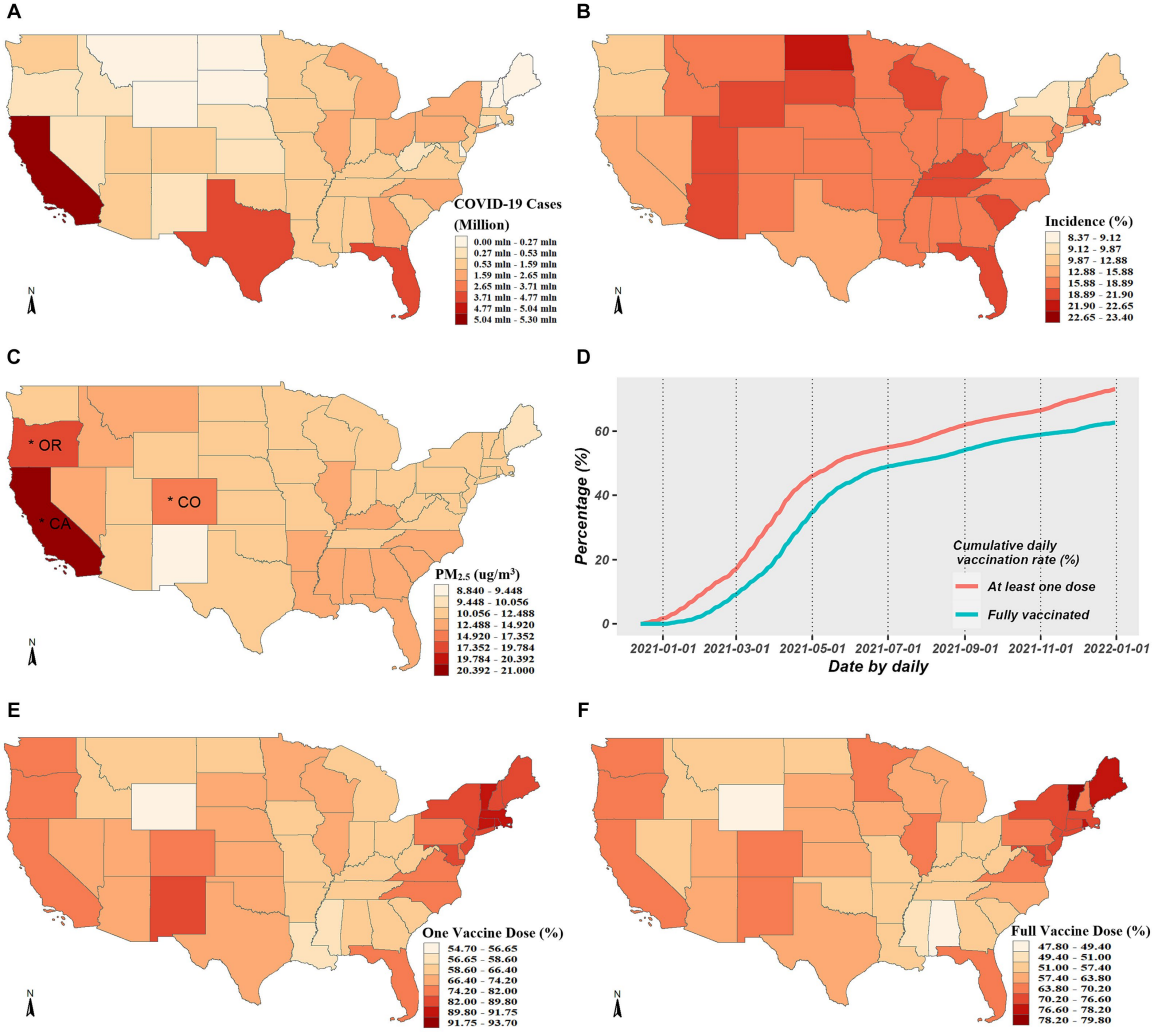
Descriptive analysis for COVID-19 cases, PM_2.5_ exposure and vaccination rates. **(A,B)** Exhibits the spatial distributions of the cumulative COVID-19 cases and incidence from April 1, 2020 to December 31, 2021, respectively. **(C)** Exhibits spatial distribution of average daily PM_2.5_ concentrations across the studied period, where the asterisks mark the three states with the highest concentrations. **(D)** Exhibits the temporal trends of vaccination rates. **(E,F)** Exhibit the spatial distributions of the cumulative vaccination rates for one or full dose, respectively.

During the period spanning from April 1, 2020 to December 31, 2021, a total of 52,250,191 COVID-19 cases were officially diagnosed across 49 states. The regional distribution of the cumulative COVID-19 cases is shown in Figure 1A. The highest cumulative instances were found in the top three states: Texas, California, and Florida. According to Figure 1B, the average COVID-19 incidence was about 16.63%. The COVID-19 incidence varied from 8 to 23% in spatial comparisons across the United States. The majority of central and eastern states had high incidences, whereas few northeastern states, Washington, and Oregon had low incidences. The state-specific average concentrations of PM_2.5_ ranged from 8.84 μg/m^3^ to 21.00 μg/m^3^, seen in Figure 1C which shows the spatial distribution of PM_2.5_ concentrations.

The average coverage rate as of December 31, 2021, was up to 72.23% for PP1V and up to 61.70% for PPFV. The whole population’s growing vaccination coverage rate over time is depicted in Figure 1D, and the spatial distributions in PP1V and PPFV at the end of 2021 are shown in Figures 1E,F. Given the significant variations in vaccination rates among states, exploring the modification role of vaccination on the PM_2.5_-COVID-19 association is valuable.

### 4.2 Spatially smoothed PM_2.5_-COVID-19 association

**Figure 2:**
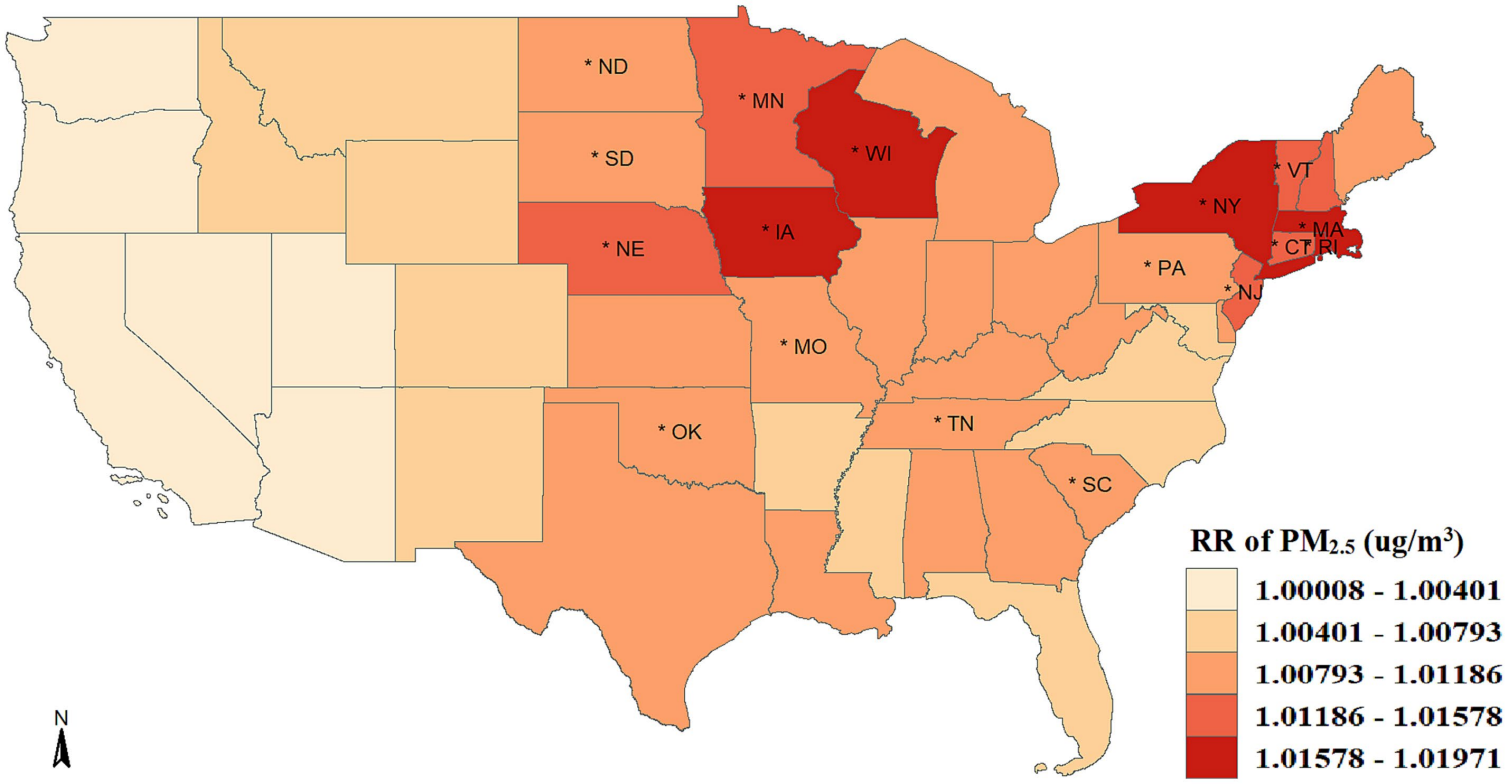
Spatial distribution of the association between PM_2.5_ exposure and COVID-19 risk. The asterisk (*) indicates that the relative risk (RR) is statistically significant (*p* < 0.05).

Figure 2 shows the spatially smoothed association between short-term exposure to PM_2.5_ and COVID-19 incidence. In general, the PM_2.5_-COVID-19 association in all states were positive, with risk ratio (RRs) over than 1. The COVID-19 incidence increased by 0.92% (95%CI: 0.63–1.23%) on average with a 1 μg/m^3^ rise in PM_2.5_ concentration. Moreover, there was significant spatial heterogeneity in the association intensity. The increasing percentage of confirmed COVID-19 cases across 49 states varied from 0.008 to 1.971% when there was a 1 μg/m^3^ increase in PM_2.5_ concentration. While all the western states showed weak associations and lost statistical significance, the majority of middle-northern and northeastern states presented significant strong associations. Specifically, mostly states with strong associations exhibited a clustering in spatial location, i.e., Vermont, Connecticut, Massachusetts, Rhode Island, New York, and New Jersey aggregated in the northeast; and Minnesota, Nebraska, Wisconsin, and Iowa aggregated in the middle-northern regions.

**Figure 3:**
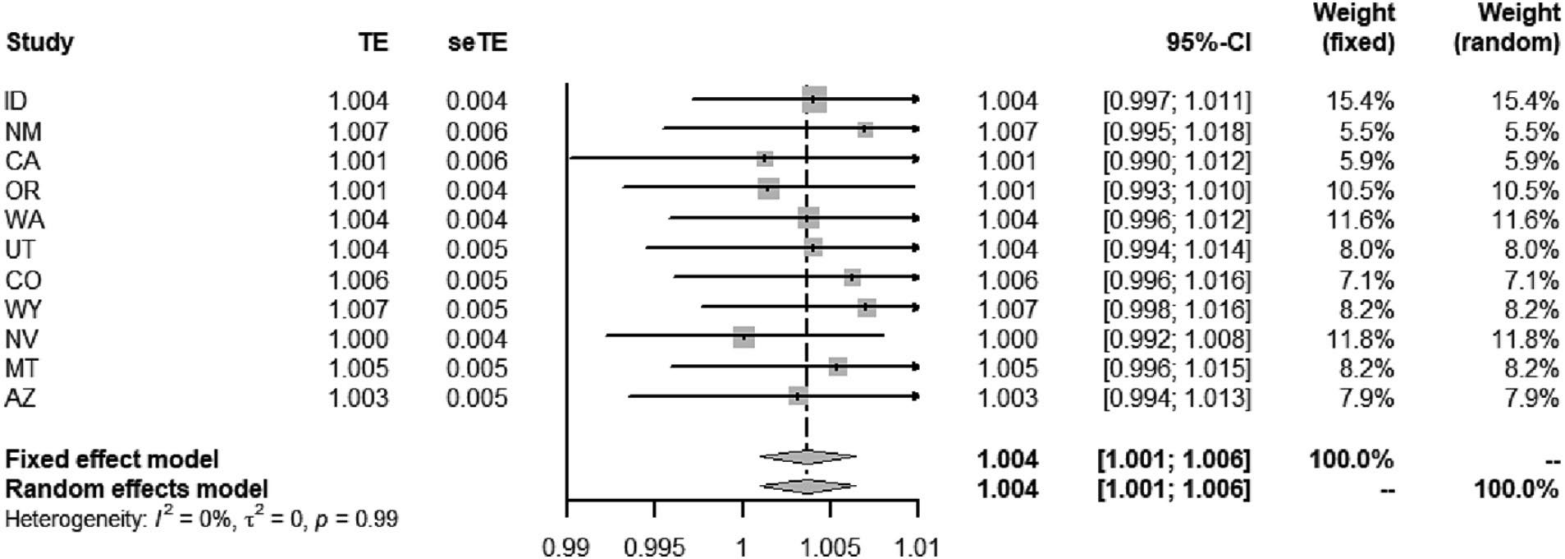
Meta-analyses of the effect of short-term PM_2.5_ exposure on the risk of COVID-19 incidence in the western states.

Additionally, although the PM_2.5_-COVID-19 associations were weak and not statistically significant in all the western states, this does not imply the disappearance of this association. The deficiency of statistical power in these local states might be the cause of insignificance. Furtherly, we performed a meta-analysis to synthesize the insignificant association in the western states, and discovered that the statistical significance of association remained, with the RR value being 1.004 (95%CI: 1.001–1.006, *p* < 0.001), seen in Figure 3. This result implies that the adverse short-term impact of PM_2.5_ on COVID-19 risk cannot be ignored in the western United States.

To evaluate the rationale of taking spatial dependence into account when utilizing the LCAR-based analytical strategy, we used the traditional two-stage strategies based on meta-analysis and stratified-analysis as reference. The former strategy holds an assumption that the associations in all states share a similarity regardless of their locations; The latter assumes no between-state similarity in the associations. We employed the commonly used model selection criteria, such as the deviance information criterion (DIC) (38) and logarithmic score (LS) (39) to assess model performance. A lower value for each index indicates improved model performance. Results showed that the LCAR-based strategy performed the best, with DIC values of −290.06, −295.86, and − 258.44, and LS values of −142.72, −147.00, −91.02 for meta-analysis, LCAR, and the stratified analysis, respectively.

### 4.3 Time-varying PM_2.5_-COVID-19 association and the effect modifier

**Figure 4:**
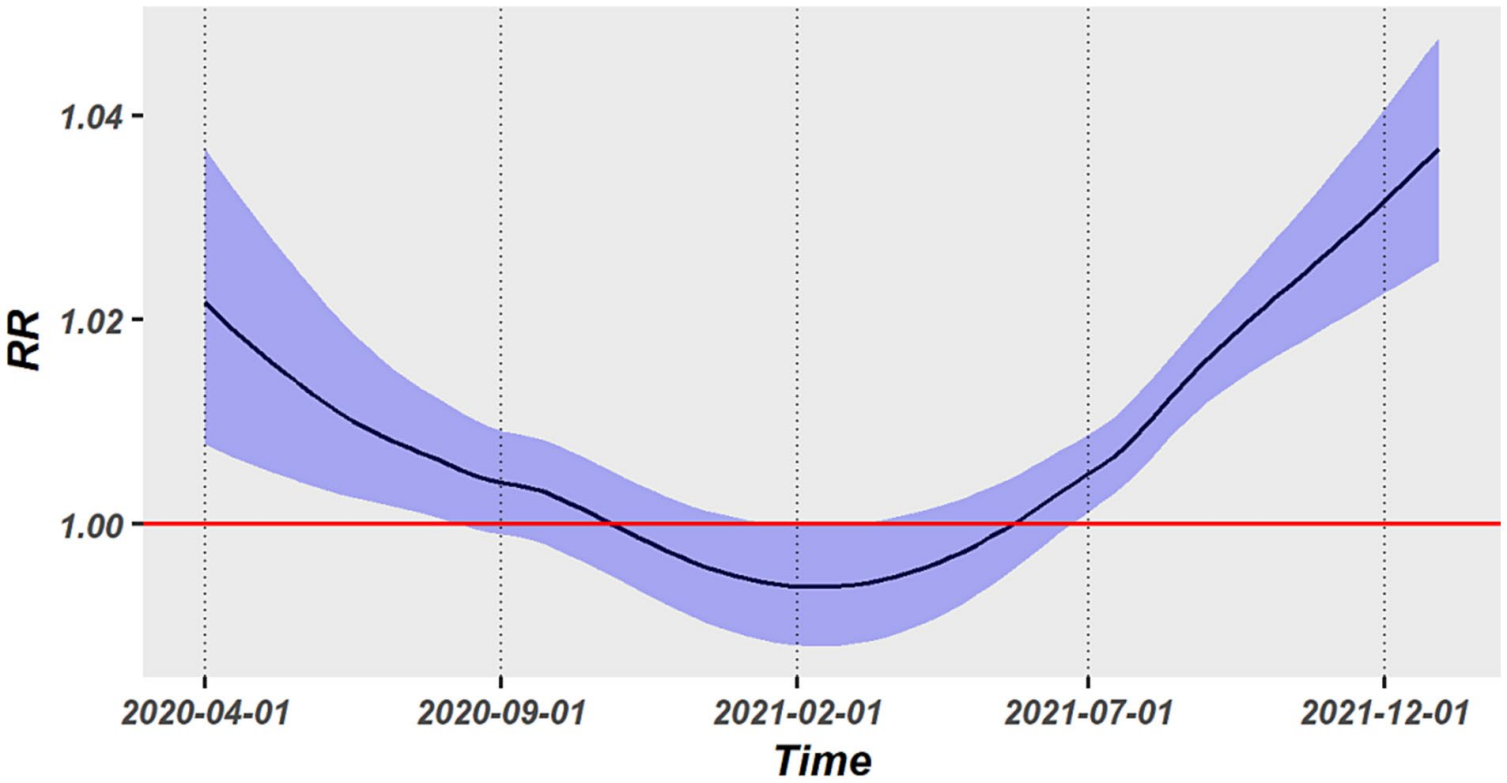
Temporal trend in the association between PM_2.5_ exposure and COVID-19 risk.

As illustrated in Figure 4, we obtained the average associations across all states at each time point by utilizing the time-varying strategy. The association presented a U-shaped temporal change, with a reduction during the early pandemic stage of COVID-19 (April 2020–February 2021) and a progressive increase from February 2021 to December 2021. The largest values of RR in the early and latter epidemic stage were, respectively, up to 1.022 (95%CI: 1.008–1.037) and 1.036 (95%CI, 1.026–1.048). The association had a lowest RR in February 2021 and was not statistically significant between September 2020 and July 2021.

**Figure 5:**
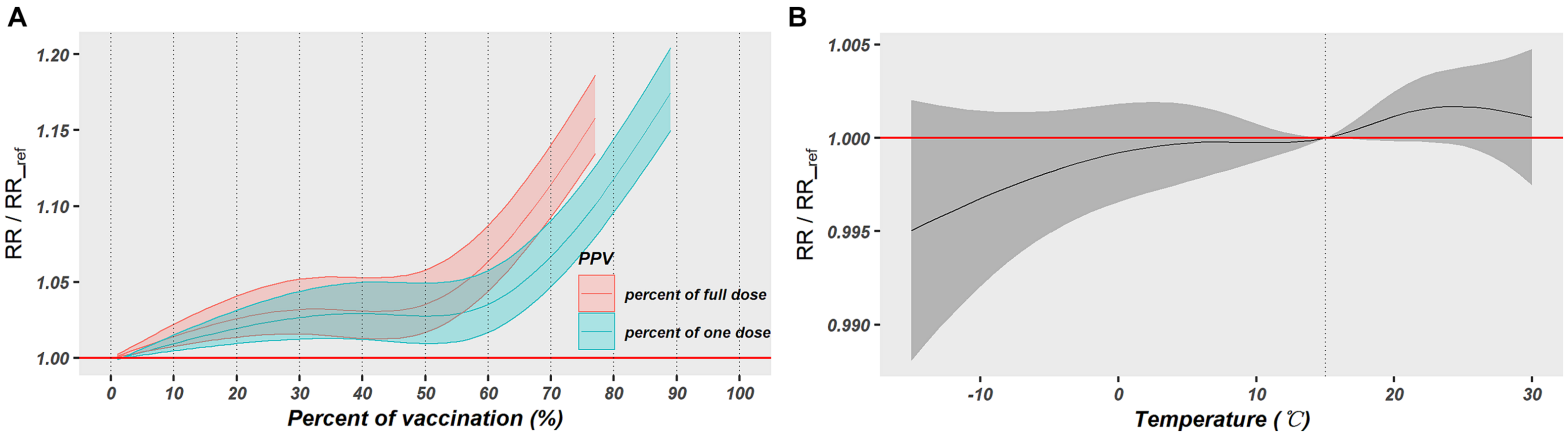
The nonlinear modification effects of vaccination and temperature on the association between PM_2.5_ exposure and COVID-19 risk. **(A)** Exhibits the modification effect of vaccination rates and **(B)** exhibits the modification effect of daily temperature.

The modification impact of vaccination and temperature on the PM_2.5_-COVID-19 association was explored by a spatiotemporal LCAR model. Both PP1V and PPFV presented significant modification effect. As shown in Table 1, the RR values of PM_2.5_ short-term exposure increased by 0.18 and 0.22%, respectively, with a 1% rise in vaccination rate, however, the temperature had no discernible modifying effect on this association. Furthermore, the nonlinear modifying effects of vaccinations and temperature on this association were also examined. Results showed that PP1V and PPFV exhibited a progressive rise within the vaccination rate of 0 and 50% in their modification effects on the PM_2.5_-COVID-19 association; however, both exhibited a faster increasing speed when vaccination rate was above 50%. Temperature did not exhibit a significant modification effect on the association, as shown in Figures 5A,B.

**Table 1:** Linear modification effect of vaccination and temperature on the association between PM_2.5_ exposure and COVID-19 risk.

Variables	Relative risk				
	Mean^1^	SD	Median	95%CI lower	95%CI higher
PP1V	1.0018	1.000097	1.0018	1.0016	1.0020
PPFV	1.0022	1.000098	1.0022	1.0020	1.0024
Temperature	1.0001	1.000068	1.0001	0.9999	1.0002

^1^This value is $e^{\hat{\theta }}$ where $\hat{\theta }$ is the estimated parameter in model (5).

To explore whether economic factors could significantly modify the PM_2.5_-COVID-19 association, we also further utilized the quarterly *per capita* GDP and monthly unemployment rate to investigate their modification effect. The results revealed that *per capita* GDP did not show a significant modification effect, which means that the level of *per capita* GDP will not have a significant effect on the PM_2.5_-COVID-19 association (RR = 1.00008, 95%CI: 0.99980–1.00036). On the other hand, the unemployment rate had a significant modified effect on the association, with a higher unemployment rate strengthening the modified effect on the association (RR = 0.99816, 95%CI: 0.99700–0.99930). Additionally, we also plotted the spatial distribution and temporal trends of economic factors, and the results are exhibited in Supplementary Figures S4–S9.

### 4.4 Sensitivity analysis

To explore whether the nonlinear association between PM_2.5_ exposure and COVID-19 incidence is consistent with the performance of linear association, the natural cubic spline function with 3 dfs was employed to reflect the nonlinear PM_2.5_-COVID-19 association. The sensitivity results, seen in Supplementary Figure S10, showed that the curve of association was approximately linear, indicating the reasonableness of the linear assumption in the main analysis.

## 5 Discussion

As far as we are aware, it is the first time to characterize the spatiotemporal heterogeneity in the association between short-term PM_2.5_ exposure and COVID-19 incidence. All United States exhibited positive associations, but which present substantially spatial heterogeneity, with the western regions showing weak associations and the middle-northern and northeastern regions showing strong associations. Moreover, the association presented a U-shaped change in the time dimension. The vaccination and unemployment rates were identified as significant effect-modifying factors for the association. Our findings play important roles on understanding the temporal trend of association between PM_2.5_ exposure and a sudden and server infectious disease like COVID-19, as well as differentiating the high-vulnerability regions and periods where and when PM_2.5_ exposure exacerbates COVID-19 infection, was made easier by. In addition, the advanced strategy also offered a cutting-edge method for exploring the spatiotemporal heterogeneity in the association between environmental factors and human health (40).

We observed positive PM_2.5_-COVID-19 associations in all the states, with an average RR of 1.009 (95%CI: 1.006–1.0123). The strength of association is in line with earlier American research, providing further evidence that the public is more susceptible to the SARS-CoV-2 infection when exposed to short-term PM_2.5_ exposure (41, 42). Regarding the spatial distribution, a considerable strong association was seen in the majority of states located in the middle-northern and northeastern regions. Such geographical aggregation indicates that type-I error from multiple tests and random spatial permutations are less likely to be the cause of the strong association. In light of this, there might be a particular epidemiological mechanism that strengthens the association in New York, Connecticut, Massachusetts, Iowa, and Wisconsin, suggesting that these states deserve a greater need for improving air pollution control and increasing government awareness of air pollutants.

Another noteworthy finding is the temporal change of association during the COVID-19 pandemic, which can be divided into three phases: early stage where the intensity of association decreases but is still significant, middle stage where the association remains stable but lacks significance, and the latter stage where the intensity of association increases. The underlying epidemiological cause of the decline and insignificant association might be attributed to public panic during the early pandemic stage of COVID-19, which considerably limited the population crowd and prompted the wearing of masks. Those behaviors could reducing the likelihood of being exposed to pollutants and the risk of contracting a virus (43). The introduction and widespread use of vaccinations, however, resulted in a decline in public awareness of self-defense and a disregard for social distancing and mask protection, which raised the probability of exposure to pollutants, thus, intensifying the association. Because of the pandemic’s enhanced vulnerability, the intensity of association was much higher at the post-pandemic period than pre-epidemic period.

The findings revealed that the uptrend section of association is precisely congruent with the introduction of vaccinations, which conforms to the result that vaccination rate was identified as a significant effect modifier in the spatiotemporal LCAR model. Therefore, the findings in our study have significant implications for public health, as they show that the degree to which contaminants affect COVID-19 is variable and closely associated to societal protection awareness and behavioral patterns of the public (44). To reduce the impact of environmental pollutants on the large-scale emerging infectious diseases such as COVID-19, the government should spend more efforts to strengthen public awareness of protection and control air pollution in the post-epidemic stages (45, 46).

Some studies indicate that economic factors also contribute significantly to the spread of the COVID-19 virus (47, 48). The expansion of economic and commercial trade activities facilitates more frequent interaction among individuals, leading to a higher risk of COVID-19 transmission (49). Furthermore, unemployment has been linked to heightened anxiety and life stress, prompting individuals to pursue new jobs prospects or seek social assistance, thereby increasing their vulnerability to COVID-19 and the potential for severe illness. The results of modified effect for *per capita* GDP and unemployment rate on the association between PM_2.5_ exposure and COVID-19 incidence showed that the level of *per capita* GDP does not significantly affect the strength of the PM_2.5_-COVID-19 association, but the increasing rate of unemployment could strengthening the modified effect on the association. However, this lack of significance may be attributed to the limited accuracy of our *per capita* GDP data, which is only available at a quarterly level. And significant modification effect of unemployment rate suggests that unemployment does not inhibit social interactions and reduce exposure to PM_2.5_. Instead, it leads to increased family burden and mental pressure, and more non-employment outings amplify the influence of PM_2.5_ exposure on COVID-19 infection (50). Through these findings, we also provided evidence that economic factors are not negligible influencing factors for the occurrence of COVID-19.

In conclusion, the spatiotemporal heterogeneity in the PM_2.5_-COVID-19 association offered important implications to the government in formulating a cost-effective policy for air pollution and infectious diseases control by using a coordinated interstate and interperiod manner. For instance, (i) strengthening the infectious disease surveillance and meteorological monitoring in high-vulnerability areas to facilitate the real-time risk assessment and early warnings; (ii) giving priority to personal protective equipment and medical resources for patients suffering from infectious diseases in high-vulnerability areas, such as Massachusetts, Iowa, New York and Wisconsin; (iii) increasing public awareness of preventive measures in high-vulnerability areas and periods; and (iv) investigating the influence of vaccination and population behavioral patterns on the risk of COVID-19, and identifying plausible epidemiological reasons to reduce the injury and damage that air pollution causes to health.

Certain limitations must be recognized because of shortcomings in the ecological design (11, 51). First, there might be measurement errors in the PM_2.5_ exposures because the state-level air pollution concentrations may not accurately represent the individual exposures that people experience in the actual world. Second, because nucleic acid detection was lacking in the early stage of the epidemic, there was an incorrect diagnosis of COVID-19 outcome. Third, during the COVID-19 pandemic, a series of SARS-CoV-2 variations were discovered, and the peak of the epidemic brought by each variant varies between different states; therefore, these variants may affect the exposure-response association.

Consequently, more validation studies using individual data or quasi-experiments, are warranted. Nevertheless, these investigations cannot be carried out in a large-scale setting due to their high cost. However, our study might offer some guidance on where and how to carry out more cost-effective studies. Research carried out in high-sensitivity areas such as New York, Massachusetts and Connecticut, for instance, is helpful to better understand the detrimental effects of PM_2.5_ exposure on the COVID-19 infection. A comparative analysis carried out concurrently in high- and low-sensitivity areas, such as California and New York, is beneficial in examining the potential causes of heterogeneous associations. Furthermore, research carried out independently at the early stages of vaccine emergence and later during vaccination can aid in investigating the reasons behind temporal heterogeneity.

## 6 Conclusion

Our study is the first one focusing on the spatiotemporal heterogeneity of association between short-term exposure to PM_2.5_ and COVID-19 occurrence. All the states in the United States exhibited positive associations. On average, a 1 μg/m^3^ increase in PM_2.5_ concentration led to an increase in COVID-19 incidence by 0.92% (95%CI: 0.63–1.23%). In the spatial dimension, a significant spatial heterogeneity was found with stronger association in the northeastern and middle-northern states and weak association in the western states. In the time dimension, a U-shaped temporal change of association was examined, with the strongest association occurring in the end of 2021 and the weakest association occurring between September 1, 2020 and July 1, 2021. Vaccination rate was identified as a significant cause for the association heterogeneity, with a stronger association occurring at a higher vaccination rate. Overall, our findings indicate that PM_2.5_ pollutants might deserve greater attention in the eastern and middle areas of the United States and in the post-pandemic era.

## Data availability statement

The original contributions presented in the study are included in the article/Supplementary material, further inquiries can be directed to the corresponding author.

## Author contributions

SL: Conceptualization, Formal analysis, Methodology, Software, Writing – original draft. SJ: Formal analysis, Investigation, Methodology, Software, Writing – review & editing. YZ: Data curation, Formal analysis, Software, Writing – review & editing. JX: Formal analysis, Software, Writing – review & editing. HZ: Conceptualization, Methodology, Supervision, Writing – review & editing. JL: Formal analysis, Software, Supervision, Writing – review & editing. DL: Methodology, Software, Writing – review & editing.

## Footnotes

^1^ https://doi.org/10.7289/V5D21VHZ

^2^ https://www.census.gov/

^3^ https://usafacts.org/topics/economy#what-is-the-current-state-of-the-us-economy
