# Supplementary material for: Exploring spatiotemporal pattern in the association between short-term exposure to fine particulate matter and COVID-19 incidence in the continental United States: a Leroux-conditional-autoregression-based strategy
Source: Front Public Health. 2023 Dec 22;11:1308775. doi: 10.3389/fpubh.2023.1308775 (PMC10768722; doi:10.3389/fpubh.2023.1308775)
Supplement: Supplementary file 1 [file Data_Sheet_1.PDF]

Exploring spatiotemporal pattern in the association between short-term exposure to fine particulate matter and COVID-19 incidence in the continental United States

1. The abbreviation for each state and the geographic locations

Table S1 The abbreviation name for each state in the continental United States

ID	State name	Abbr	ID	State name	Abbr	ID	State name	Abbr
1	Alabama	AL	19	Maryland	MD	37	Pennsylvania	PA
2	Arizona	AZ	20	Massachusetts	MA	38	Rhode Island	RI
3	Arkansas	AR	21	Michigan	MI	39	South Carolina	SC
4	California	CA	22	Minnesota	MN	40	South Dakota	SD
5	Colorado	CO	23	Mississippi	MS	41	Tennessee	TN
6	Connecticut	CT	24	Missouri	MO	42	Texas	TX
7	Delaware	DE	25	Montana	MT	43	Utah	UT
8	District of Columbia[1]	DC	26	Nebraska	NE	44	Vermont	VT
9	Florida	FL	27	Nevada	NV	45	Virginia	VA
10	Georgia	GA	28	New Hampshire	NH	46	Washington	WA
11	Idaho	ID	29	New Jersey	NJ	47	West Virginia	WV
12	Illinois	IL	30	New Mexico	NM	48	Wisconsin	WI
13	Indiana	IN	31	New York	NY	49	Wyoming	WY
14	Iowa	IA	32	North Carolina	NC			
15	Kansas	KS	33	North Dakota	ND			
16	Kentucky	KY	34	Ohio	OH			
17	Louisiana	LA	35	Oklahoma	OK			
18	Maine	ME	36	Oregon	OR			

Note: [1] District of Columbia is deemed as a special state for writing convenience.

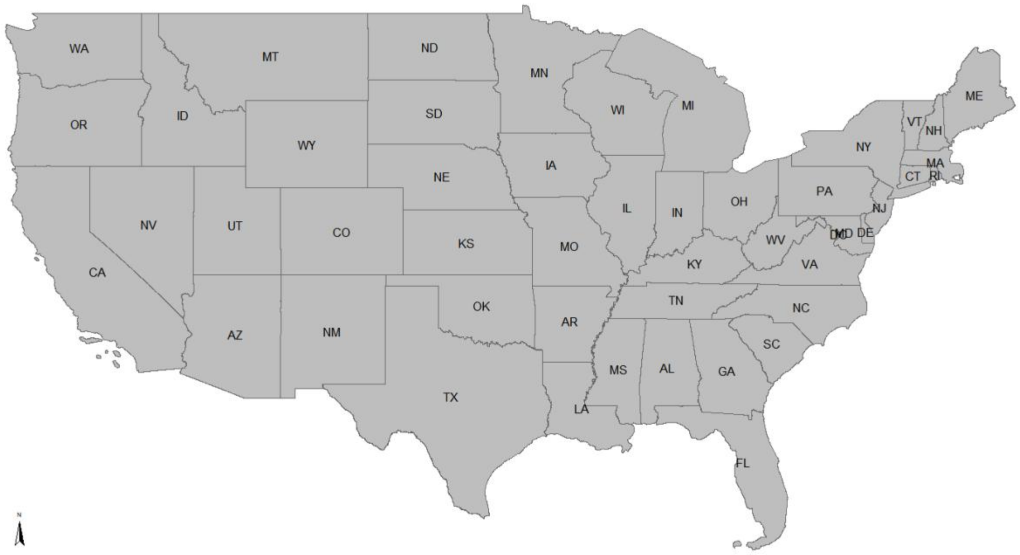

Figure S1 The geographic location for each state in the continental United States

2. Parameter selection for generalized additive models (GAMs)

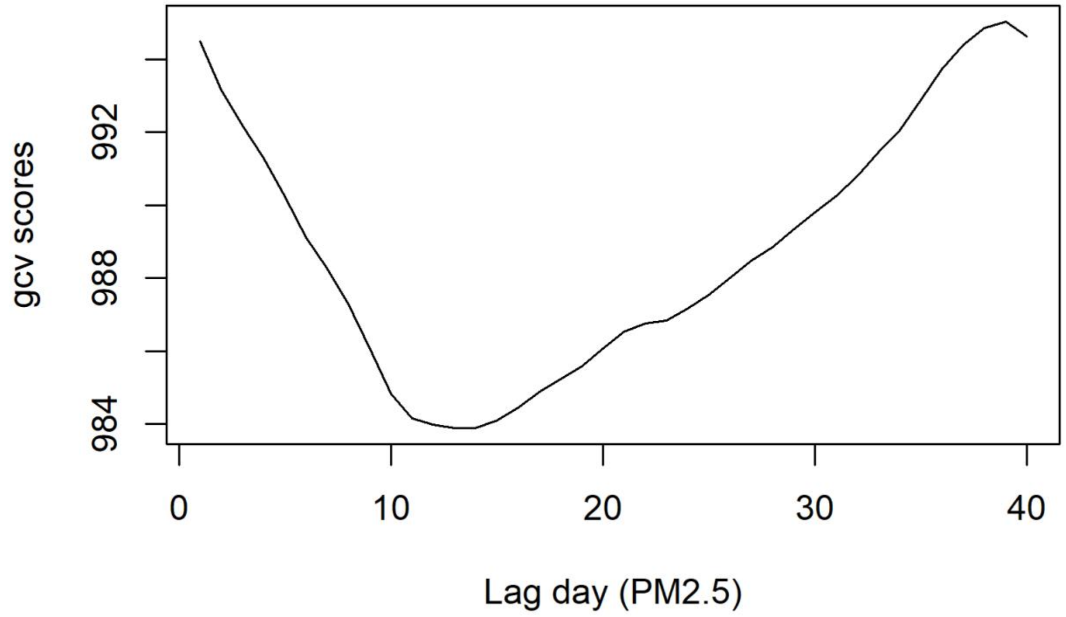

Figure S2 The generalized cross-validation scores of $PM_{2.5}$ with different lag days

Table S2 The candidate forms for the autoregression term in GAM

ID	Variable Dimension	Lag Dimension	Total *df*
**Temperature**			
1	ns[1] with *df* = 3	Exponential moving average	3
2	ns with *df* = 4	Exponential moving average	4
3	ns with *df* = 5	Exponential moving average	5
4	ns with *df* = 6	Exponential moving average	6
**Pressure**			
1	ns with *df* = 3	Exponential moving average	3
2	ns with *df* = 4	Exponential moving average	4
3	ns with *df* = 5	Exponential moving average	5
4	ns with *df* = 6	Exponential moving average	6
**Time**			
1	ns with *df* = 4 per year	NA	NA
2	ns with *df* = 5 per year	NA	NA
3	ns with *df* = 6 per year	NA	NA
4	ns with *df* = 7 per year	NA	NA
5	ns with *df* = 8 per year	NA	NA
6	ns with *df* = 9 per year	NA	NA
7	ns with *df* = 10 per year	NA	NA
8	ns with *df* = 11 per year	NA	NA
9	ns with *df* = 12 per year	NA	NA

Note: [1] ns represents that the nonlinear relationship is characterized by natural cubic spline. The numbers of daily cases with a lag of 0-14 days were used as the variable constructing the temperature and pressure term.

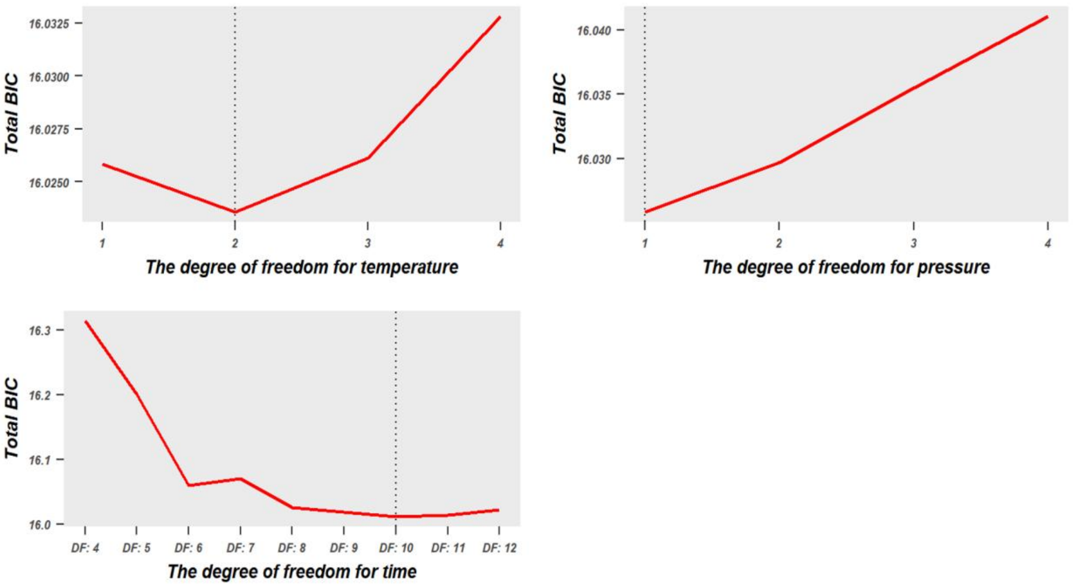

Figure S3 The performance of each candidate term. The dot line indicates the selected terms in the final models

3. Sensitivity analysis

3.1 The modified effect of economic indicators on the association

3.1.1 The modified effect of per capita GDP

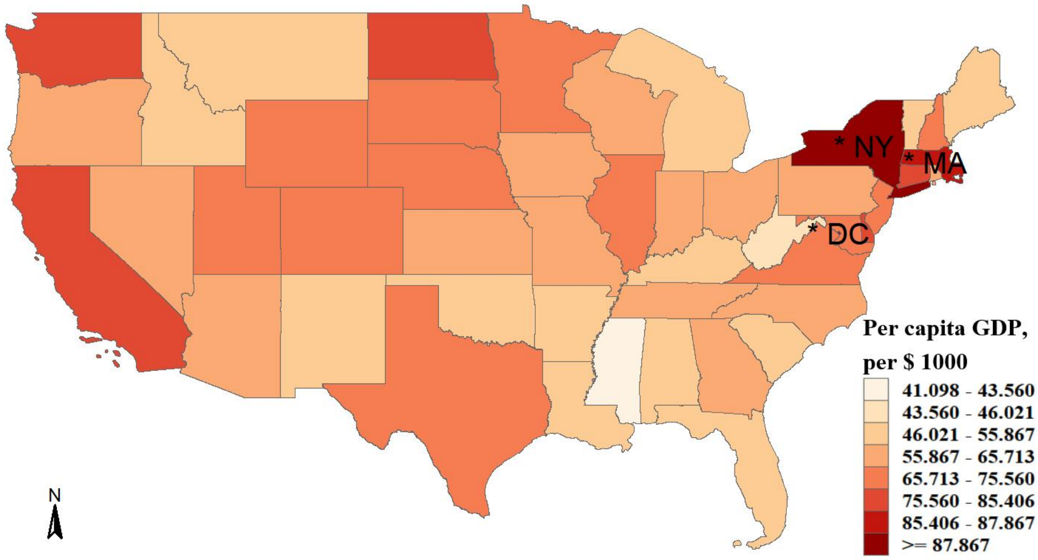

Figure S4 The spatial distributions of the average per capita GDP by state.

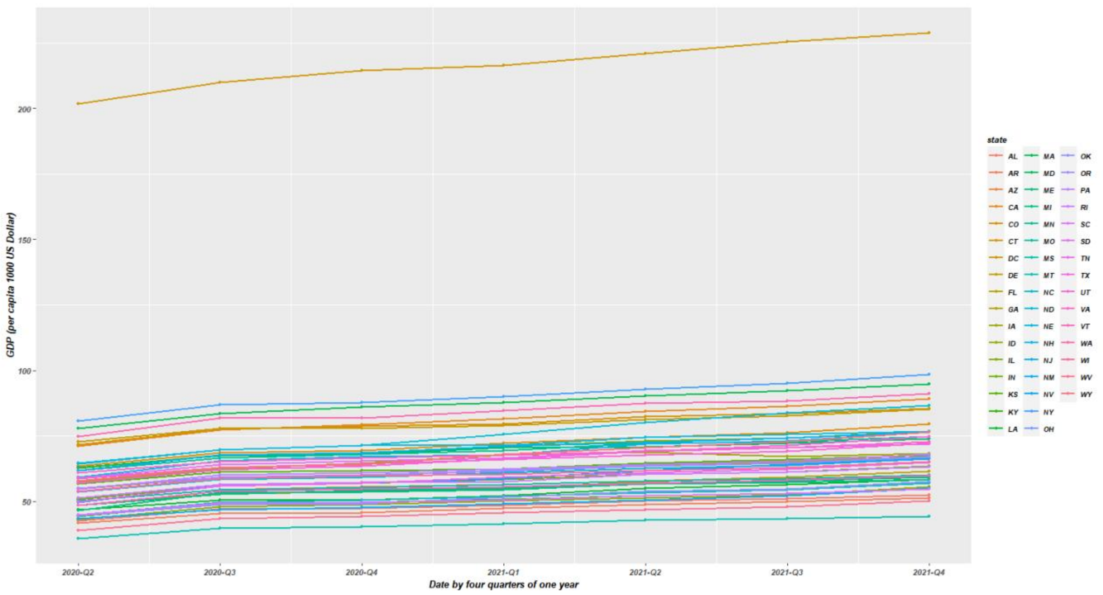

Figure S5 The temporal trends of the average per capita GDP by state.

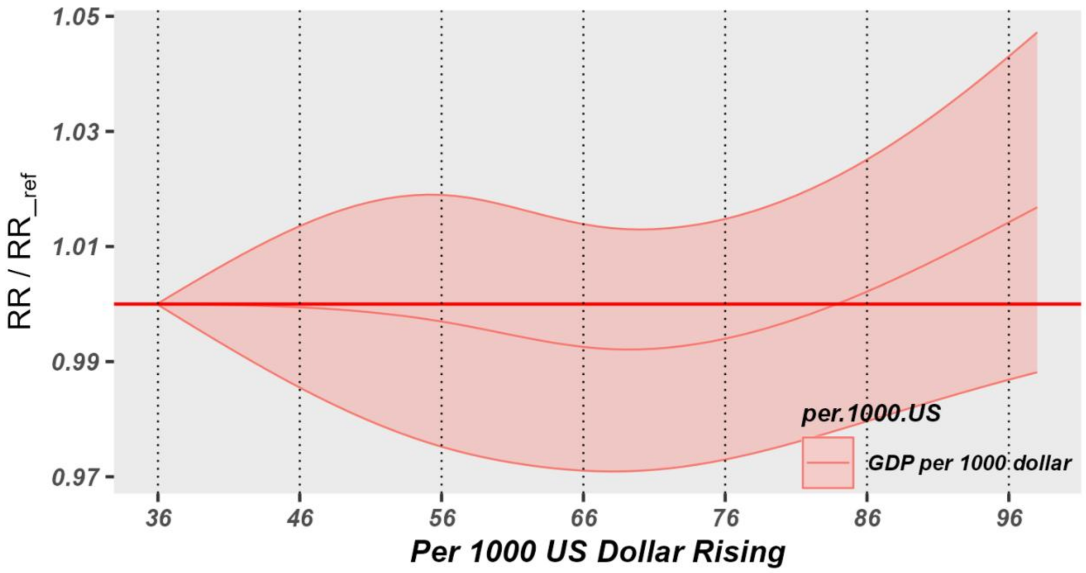

Figure S6 The nonlinear modification effect of per capita GDP on the $PM_{2.5}$-COVID-19 association.

3.1.2 The modified effect of unemployment rate

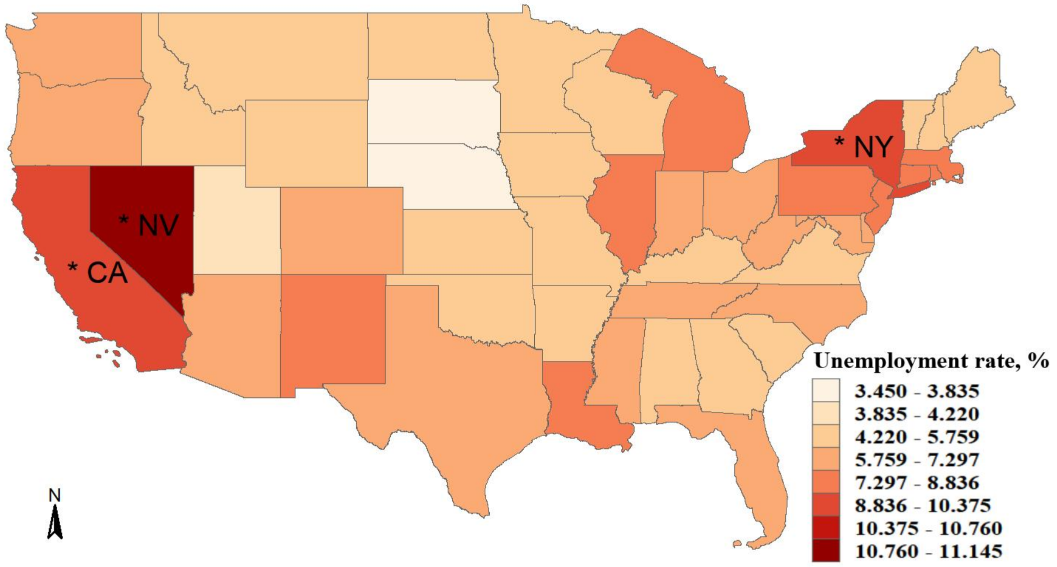

Figure S7 The spatial distributions of the unemployment rate by state.

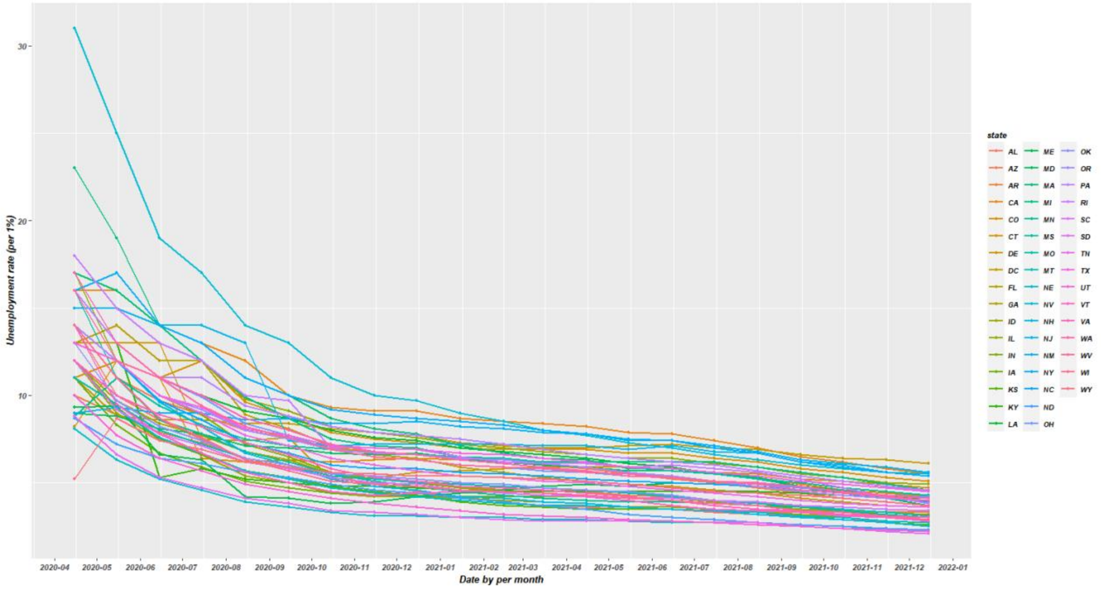

Figure S8 The temporal trends of the unemployment rate by state.

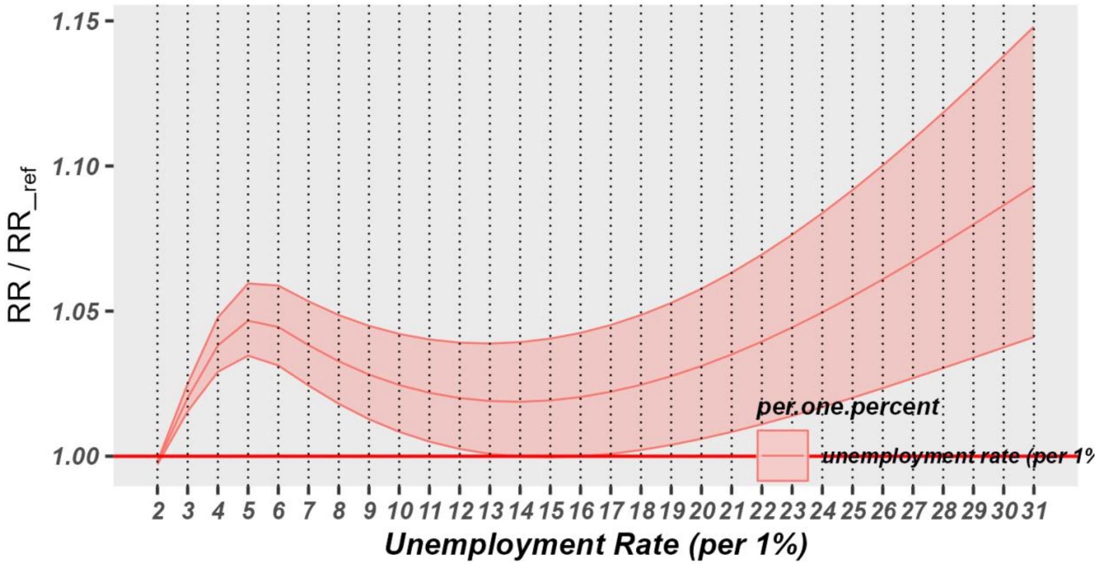

Figure S9 The nonlinear modification effect of unemployment rate on the $PM_{2.5}$-COVID-19 association.

3.3 Verification of the linear association

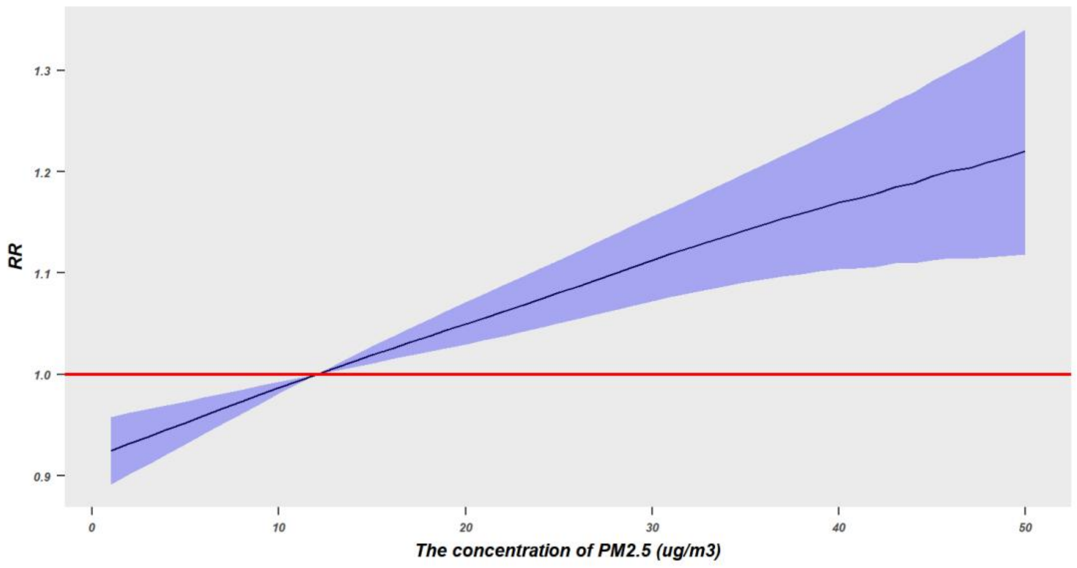

Figure S10 The nonlinear association between $PM_{2.5}$ exposure and COVID-19.
